# Identifying the role of ASPN and COMP genes in knee osteoarthritis development

**DOI:** 10.1186/s13018-019-1391-7

**Published:** 2019-10-29

**Authors:** Abhishek Mishra, Sachin Awasthi, Saloni Raj, Priya Mishra, Rajeshwar Nath Srivastava

**Affiliations:** 1 Centre for Advanced Research, King George’s Medical University, Lucknow, 226003 India; 2 Orthopedic Surgery, Dr. Ram Manohar Lohia Institute of Medical Sciences, Lucknow, 226001 India; 3 Westminster College, Salt Lake City, UT USA; 4 Department of Prosthodontics, King George’s Medical University, Lucknow, India; 5 Department of Orthopedic Surgery, King George’s Medical University, Lucknow, 226003 India

**Keywords:** Knee osteoarthritis, Gene, Gene expression, Case–control

## Abstract

**Background:**

Osteoarthritis (OA) is a common cause of musculoskeletal disability among elders and is characterized by late-onset degeneration of articular cartilage. OA affects various joints, commonly hand, knee, and hip, with clinical features that are unique to each joint. This study was initiated to identify and evaluate the role of the ASPN and COMP genes in the development of knee OA.

**Methods:**

A case–control study was carried out involving 500 cases with knee OA (diagnosed by the American College of Rheumatology) and an equal number of healthy controls. Blood was drawn for genomic DNA isolation. PCR-RFLP and TaqMan assay methods were used to identify the SNPs. mRNA and protein expression of genes were carried out in peripheral blood lymphocytes (PBLs) by RT-PCR and Western immunoblotting. The data obtained were analyzed for the statistical significance between control and case groups.

**Results:**

The variant genotype of ASPN and COMP genes was found to be present at a relatively higher frequency in cases than controls. RT-PCR and immunochemical studies revealed increased mRNA and protein expression of such gene in PBLs isolated from cases of knee OA as compared to healthy control.

**Conclusion:**

The allelic alteration in ASPN and COMP genes in knee OA cases points to the role of these genes in the development of knee OA. Further, increased mRNA and protein expression of ASPN and COMP in peripheral blood samples of patients with the disease suggest that expression profile of candidate gene could be used as a biomarker for predicting the development and progression of knee OA.

## Background

Osteoarthritis (OA) is one of the most prevalent chronic diseases and a leading cause of disability among the elderly [1]. It is characterized by a gradual loss of articular cartilage in the joint. It is a complex disease entity that is difficult to diagnose and define; clinically, the condition is characterized by joint pain, tenderness, limitation of movement, crepitus, occasional effusion, and variable degrees of local inflammation [2–4]. Knee OA causes loss of joint function, leading to reduced activity, and decreases the quality of life. In addition to an impact on health, the disease also has economic and social implications [4, 5]. Epidemiological and genetic studies have demonstrated that genetics is an important factor in the development and progression of knee OA [6]. Studies have shown that genetic polymorphisms in ASPN and COMP have an effect on knee OA and that the expression of these genes varies between populations (Japanese versus Caucasian) [7, 8].

Genetic studies of ASPN gene polymorphism have demonstrated an association between ASPN and various bone and joint diseases, including knee osteoarthritis, rheumatoid arthritis, and lumbar disc disease [9]. Asporin is an extracellular protein belonging to the small leucine-rich proteoglycan family of ASPN gene. These proteins can bind and regulate the activity of the cartilage growth transforming growth factor-β (TGF-β) [10]. Asporin is upregulated in disease states. It binds to various growth factors, including TGF-β and BMP-2, and negatively regulates their activity. By inhibiting the binding of TGF-β1 to its type II receptor, Asporin forms a functional feedback loop with TGF-β1 and regulates its chondrogenic potential [11]. As an extracellular, tissue-specific protein, Asporin represents a promising target for pharmacogenomics approaches to common bone and joint diseases.

Previous studies have reported that COMP affects the factor that plays a role in the development of knee OA [8, 12].COMP is a major component of the extracellular matrix and is thought to play a major role in the development and homeostasis of the cartilage [12]. It is abundantly expressed in the chondrocyte extracellular matrix and is also found in bone, tendon, ligament, synovium, and blood [13]. A previous study also reported that COMP play a role in the development of such disease [14]. Osteoarthritis is due to an imbalance between cartilage degradation and synthesis and causes the cartilage layer to become thinner [15]. COMP fragments are released into the joint fluid following injury and during the early stages of osteoarthritis, and therefore, COMP is considered a marker for cartilage degradation [16]. It has been shown that decreased cartilage volume is related to the onset of knee OA, and to compensate for the diminished cartilage space, the bone beneath thickens and spreads out to form knobby outgrowths (osteophytes) [15, 16].

It is difficult to investigate the functional activity of these candidate genes in the cartilage due to ethical issues related to obtaining cartilage. Expression of these candidate genes in peripheral blood lymphocytes (PBLs) could potentially be used as a biomarker for evaluating the development of knee OA [17]. Recently, efforts have been made to develop biomarkers that could identify the risk factors involved in knee OA and monitor the progression of the disease. Functional studies would help in identifying the role of genes involved in predicting risk to knee OA. Monitoring the expression of candidate gene in PBLs would lead to the development of a biomarker that may further support the genotype study and provide the functional role of these genes involved in predisposing the individual to the risk of OA.

However, limited information is available on the association of ASPN and COMP with knee OA in the Caucasians and Oriental populations. There is no comparable study in the Indian population. The present study was therefore initiated to investigate the association of polymorphism in ASPN and COMP gene with knee OA in the north Indian population. To study the functional role of ASPN and COMP in knee OA, the expression of these genes in knee OA patients was investigated.

## Material and methods

A case–control study of 500 controls and 500 cases of knee OA were carried out in this study. The patients fulfilled the American College of Rheumatology (ACR) clinical and radiographic criteria of knee OA. The controls did not show any evidence of knee OA. Radiographic findings of knee OA were classified into mild (KL grade 2), moderate (KL grade 3), and severe (KL grade 4). Symptoms related to knee OA were assessed with the knee-specific WOMAC index (Bellamy, 1989) [16], which assesses pain (five items), stiffness (two items), function (17 items), and interpretation response on a 5-point scale (0, none; 1, slight; 2, moderate; 3, severe; 4, extreme). Knee pain was also assessed using VAS, where higher scores indicate worst status. These patients were profiled for demographic, clinical, radiological, and biochemical features. The protocol for research work was approved by the Human Ethics Committee of King George’s Medical University, Lucknow. The protocol conforms to the provisions of the Declaration of Helsinki in 1995. Informed consent was obtained from the study subjects for inclusion in the study. Before the collection of blood samples, subjects were anonymized. The control and cases were asked to fill out the detailed questionnaire regarding their occupation, socioeconomic status, medical history, lifestyle habits, etc.

### DNA isolation and genotyping studies

Approximately 1 ml of blood was collected into citrate-containing tubes from all the subjects. DNA was isolated from whole blood with the Flexi Gene DNA kit (Qiagen, CA) following the manufacturer’s protocol.

### Detection of cartilage-oligomeric matrix protein polymorphisms

The functionally important cartilage-oligomeric matrix protein (COMP) gene polymorphism (HpyCH4IV (rs34467947) &c279C/A) was detected using designed restriction fragment length polymorphism (RFLP). PCR reaction resulted in a 130-bp product for HpyCH4IV and 273 bp for c279C/A polymorphism. PCR products (10 μl) were digested with 10 U of HpyCH4IV (New England Biolabs) and NciI restriction enzyme (MBI Fermentas, Germany) to identify the presence of polymorphic sites in the COMP gene.

Digestion of 130 bp PCR product of HpyCH4IV into two fragments of 75 bp and 55 bp indicates the presence of CC genotype of HpyCH4IV. The presence of fragments of three sizes (130 bp, 75 bp, and 55 bp) was indicative of the CT genotype while the undigested 130 bp PCR fragment was indicative of TT genotype of HpyCH4IV. Similarly, digestion of 273 bp PCR product of c279C/A polymorphism into three fragments of 148 bp, 110 bp, and 15 bp indicates the presence of CC genotype of c279C/A polymorphism. The results of PCR-RFLP were validated through DNA sequencing.

The SNPs of ASPN rs3739606 and rs331377 were detected by TaqMan assay (C__15754762_10) in 96-well plates after optimization for each primer set (ABI, Foster, CA, USA). The reaction was performed using an ABI 7900 HT fast real-time PCR system (50 °C for 2 min, 95 °C for 10 min, 95 °C for 15 s, and 60 °C for 1 min, for 40 cycles) and analyzed using an SDS RQ manager 1.2 software according to the manufacture’s instruction.

### RNA isolation and gene expression analysis by real-time quantitative reverse transcription PCR (real-time qRT-PCR)

Total RNA was extracted from whole blood with TRI-BD (Sigma, USA) according to the manufacturer’s protocol. The protocol utilizing TRI-BD reagent, a monophasic solution of phenol and guanidiumisothiocyanate, is an improvement of the single-step RNA isolation developed by Chomczynski and Sacchi [18]. The ASPN and COMP mRNA expression were quantified using reverse transcriptase-PCR. Equal amounts of RNA were reverse transcribed using the superscript first-strand cDNA synthesis kit with Oligo-dT (Invitrogen, USA) and diluted in nuclease-free water (Ambion) to a final concentration of 5 ng/μl. Expression of house-keeping gene β-actin served as a control to normalize values. Targets were detected and quantified in real time using the ABI Prism 7900 sequence detector system (PE Applied Biosystems; Foster City, CA, USA) and SYBR green chemistry (Applied Biosystems, USA). Relative expression was calculated using the ΔΔCt method.

### Isolation of lymphocytes and protein expression by Western blot

Lymphocytes were isolated from the blood by the method describe previously by Dey et al. [19]. In brief, after sonication, peripheral blood lymphocytes (PBLs) isolated from case and control were used for immunoblotting. Protein content of the case and control samples was estimated by the method describe by Lowry [20]. The blood lymphocytes were separated by sodium dodecyl sulfate–polyacrylamide gel electrophoresis (SDS–PAGE; 3% stacking gel and 7.5% separating gel) and electroblotted on Immobilon-P membrane (Millipore, USA). The membranes were incubated with the primary antibodies raised against ASPN [21] and COMP [22] (1:1000 dilution) in 5 ml of phosphate-buffered saline containing 0.02% Tween-20 and 0.02% sodium azide, PBST for 3 h at room temperature. The membranes carrying lymphocytes were incubated with 1:2000 dilution of rabbit anti-rabbit IgG-horseradish peroxidase (secondary antibody). Following incubation, membranes were washed five times with PBST (5–10 min each) and then processed for detection with chemiluminescence substrate. The membranes were visualized on VERSA DOC Imaging System (Model 1000, Bio-Rad, USA) and quantitative analysis using Quantity One Quantitation software (Bio-Rad). Prior to studying protein expression of these genes, normalization of proteins in the lymphocytes was carried out using beta-actin antibody.

### Statistical analysis

Genotype or allele frequencies of ASPN and COMP among cases and controls were determined for Hardy–Weinberg equilibrium (HWE) using standard chi-square statistics. Using binary logistic regression models, the relationship of ASPN and COMP gene polymorphisms with risk of knee OA was determined. Student’s *t* test was employed to calculate the statistical significance between control and case groups. All statistical analyses were performed with the SPSS software package (version 16.0 for Windows; SPSS Chicago, IL). The power of the present test results was > 80% with 95% significance levels analyzed by power genetic association analysis software (http://dceg.cancer.gov/bb/tools/pga).

## Result

The main characteristic of the study population is summarized in Table 1. The distribution of genotype of ASPN and COMP gene is summarized in Tables 2 and 3. The genotypes of ASPN and COMP in controls were found to be in Hardy–Weinberg equilibrium (HWE). Table 2 of ASPN gene shows that the variant genotype (TT of rs3739606 and GG of rs331377) frequency of ASPN was increased in the case group as compared to the control group. The genotype frequency of variant genotype (TT) of rs3739606 was increased in cases than controls. This increase in the frequency of variant genotype was significantly associated with 1.64 fold increase risk to knee OA (O.R. − 1.64; 95% CI − 1.00–2.69, *p* value = 0.046). On gender-wise stratification, no significant association was observed in females and males; however, an increased odd was found in male cases compared to female. An overrepresentation of variant genotype (GG) of ASPN (rs331377) gene is observed in cases. When the cases were stratified on the basis of gender, the frequency of GG genotype was more in male cases. Similarly, an overrepresentation of variant genotype of COMP gene is reported in cases. Table 3 summarizes the genotype distribution of HpyCH4IV polymorphisms (rs34467947) of COMP gene in the knee OA cases and the controls. As evident from the table, the frequency of TT and CT genotype was found to be higher in cases compared to controls. A slightly increased OR, though not statistically significant, was observed when the frequency of TT genotype in cases was compared with controls (OR 1.78; 95% CI 0.51–6.13). No risk was also observed on comparing the frequency of the CT genotype of cases compared with controls (OR 1.23; 95% CI 0.74–2.04). Similar pattern was also observed when the frequency of TT and CT genotype of HpyCH4IV polymorphisms in women and men patients were compared with the respective controls. Percentage of risk allele T was not much higher in cases compared to controls. The frequency of variant allele T was found to be increased in both male and female cases compared to controls (Table 3). Polymorphism in COMP (c279C/A) gene could not be detected, as the frequency of the mutant allele is very rare in the Indian population.

**Table 1:** Characteristics of the study population

	Controls (500)	Cases (500)	*p* value
Age (mean ± SD, years)	55.26 ± 8.26	55.86 ± 8.89	0.277
Female age (mean ± SD, years)	55.52 ± 9.04	55.67 ± 8.58	0.839
Male age (mean ± SD, years)	54.95 ± 8.09	56.15 ± 9.33	0.155
BMI (mean ± SD, kg/m^2^)	23.39 ± 2.39	25.41 ± 3.23	> 0.001*
Female BMI (mean ± SD, kg/m^2^)	23.59 ± 2.48	25.81 ± 3.55	> 0.001*
Male BMI (mean ± SD, kg/m^2^)	23.14 ± 2.25	24.84 ± 2.61	> 0.01*
Females (*n* %)	276 (55.2%)	295 (59.0%)	
KL grade 2/3/4	224 (44.8%)	205 (41.0%)	
VAS (mean ± SD)	–	6.14 ± 1.13	
Total WOMAC (mean ± SD)	–	35.47 ± 8.84	

*BMI* body mass index, *KL grade* Kellgren–Lawrence Grading Scale, *VAS* visual analog scale, *WOMAC* The Western Ontario and McMaster Universities Osteoarthritis Index

*p < 0.05 is considered statistically significant

**Table 2:** Genotype association between SNPs in ASPN gene and knee osteoarthritis

	Genotype	All subjects			Women			Men		
		Control (%)	Case (%)	OR (95% CI), *p* value	Control (%), 276	Case (%), 295	OR (95% CI), *p* value	Control (%), 224	Case (%), 205	OR (95% CI), *p* value
Genotype	3739606									
	GG	261 (52.2)	238 (47.6)	1.00 (ref)	139 (50.36)	139 (47.79)	1.00 (ref)	122 (54.46)	99 (48.29)	1.00 (ref)
	GT	209 (41.8)	217 (43.4)	1.13 (0.87–1.47), 0.325	119 (43.11)	129 (43.72)	1.06 (0.75–1.50), 0.703	90 (40.17)	88 (42.92)	1.20 (0.81–1.79), 0.355
	TT	30 (6.0)	45 (09.0)	1.64 (1.00–2.69), 0.046*	18 (6.52)	27 (8.47)	1.36 (0.71–2.62), 0.341	12 (5.35)	18 (8.78)	1.84 (0.85–4.02), 0.117
Allele	G	731 (73.1)	693 (69.3)	1.00 (ref)	397 (71.92)	411 (69.66)	1.00 (ref)	334 (74.53)	286 (69.75)	1.00 (ref)
	T	269 (26.9)	307 (30.7)	1.20 (0.99–1.46), 0.060	155 (28.07)	179 (30.33)	1.11 (0.86–1.44), 0.401	114 (25.44)	124 (30.24)	1.27 (0.94–1.71), 0.116
Genotype	Rs 331377									
	AA	201 (40.2)	183 (36.6)	1.00 (ref)	114 (41.30)	111 (37.62)	1.00 (ref)	87 (38.83)	72 (35.12)	1.00 (ref)
	AG	154 (30.8)	166 (33.2)	1.18,(0.88–1.59), 0.264	86 (31.15)	96 (32.54)	1.14 (0.77–1.69), 0.493	68 (30.35)	70 (34.14)	1.24 (0.78–1.96), 0.349
	GG	145 (29.0)	151 (30.2)	1.14 (0.84–1.54), 0.385	76 (27.53)	88 (29.83)	1.18 (0.80–1.78), 0.368	69 (30.80)	63 (30.73)	1.10 (0.69–1.75), 0.677
Allele	A	556 (55.6)	532 (53.2)	1.00 (ref)	314 (56.88)	318 (53.89)	1.00 (ref)	242 (54.01)	214 (52.19)	1.00 (ref)
	G	444 (44.4)	468 (46.8)	1.10 (0.92–1.31), 0.281	238 (43.11)	272 (46.10)	1.12 (0.89–1.42), 0.310	206 (45.98)	196 (47.80)	1.07 (0.82–1.40), 0.593

*OR* odds ratio, *95% CI* 95% confidence interval, *ref* reference category

*p < 0.05 is considered statistically significant

**Table 3:** Genotype association between SNP in COMP (HpyCH4IV_rs34467947) gene and knee osteoarthritis (KOA)

		All subjects			Women			Men		
		Control (%)	Case (%)	OR (95% CI), *p* value	Control (%), 276	Case (%), 295	OR (95% CI), *p* value	Control (%), 224	Case (%), 205	OR (95% CI), *p* value
Genotype	HpyCH4IV-rs34467947									
	CC	467 (93.4)	458 (91.6)	1.00 (ref)	259 (93.84)	272 (92.20)	1.00 (ref)	208 (92.85)	186 (90.73)	1.00 (ref)
	CT	29 (5.8)	35 (7.0)	1.23 (0.74–2.04), 0.423	14 (5.07)	18 (6.10)	1.22 (0.59–2.51), 0.580	15 (6.69)	17 (8.29)	1.26 (0.61–2.60), 0.519
	TT	4 (0.8)	7 (1.4)	1.78 (0.51–6.13), 0.351	3 (1.08)	5 (1.69)	1.58 (0.37–6.70), 0.526	1 (0.04)	2 (0.9)	2.23 (0.20–24.86), 0.501
Allele	C	963 (96.3)	951 (95.1)	1.00 (ref)	532 (96.37)	562 (95.25)	1.00 (ref)	431 (96.20)	389 (94.87)	1.00 (ref)
	T	37 (3.7)	49 (4.9)	1.34 (0.86–2.07), 0.185	20 (3.62)	28 (4.74)	1.32 (0.73–2.38), 0.344	17 (3.79)	21 (5.12)	1.36 (0.71–2.63), 0.345

*OR* odds ratio, *95% CI* 95% confidence interval, *ref* reference category

**Fig. 1:**
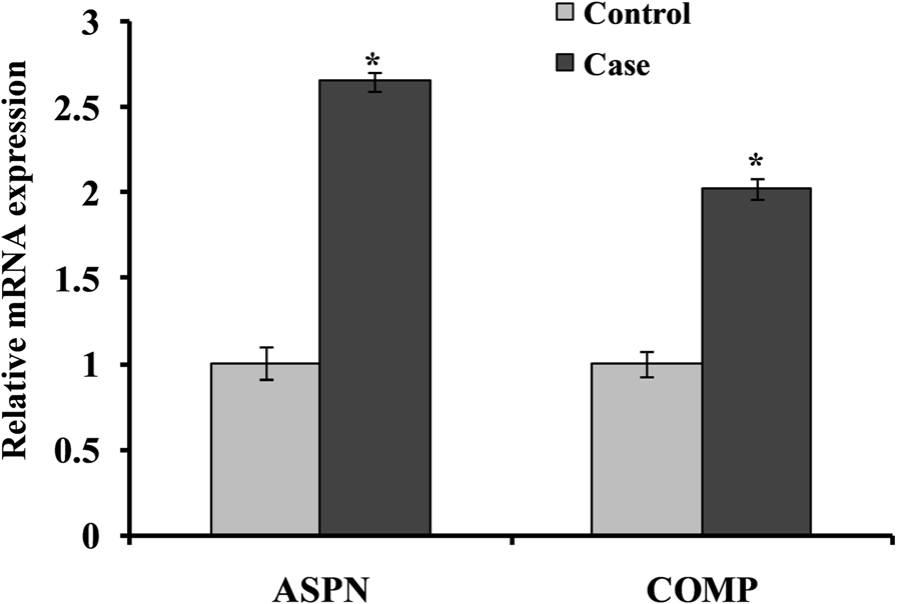
Quantitative real-time PCR analysis was performed for relative mRNA expression of genes that were involved in the cartilage catabolism in control and case study. Quantitative analysis suggested that expression of ASPN and COMP genes upregulated in case group compared to their respective control. β-Actin served as housekeeping gene for normalization. Values are expressed as mean ± SEM (*n* = 24). *p < 0.05 vs control

Quantification of ASPN and COMP gene expression by RT-PCR revealed that ASPN and COMP were expressed in freshly prepared blood lymphocytes isolated from healthy individuals (Fig. 1). The mean number of copies detected for ASPN and COMP gene (expressed as mRNA detected/5 μg total RNA) was found to be increased in cases. The mRNA expression of housekeeping gene (β-actin) was used as an endogenous control. The uniform expression of β-actin in all the samples (control and knee OA case) further confirmed the integrity of RNA used in assays. A typical basal expression profile for ASPN was about 2.64-fold upregulated. Increased ASPN mRNA expression was observed in cases compared to controls (Fig. 1). RT-PCR data further revealed that mRNA expression of COMP in knee OA patients upregulated 2.01-fold in blood lymphocytes compared to controls (Fig. 1). Student *t* test analysis of RT-PCR data has also shown a statistically significant effect (*p* > 0.05).

**Fig. 2:**
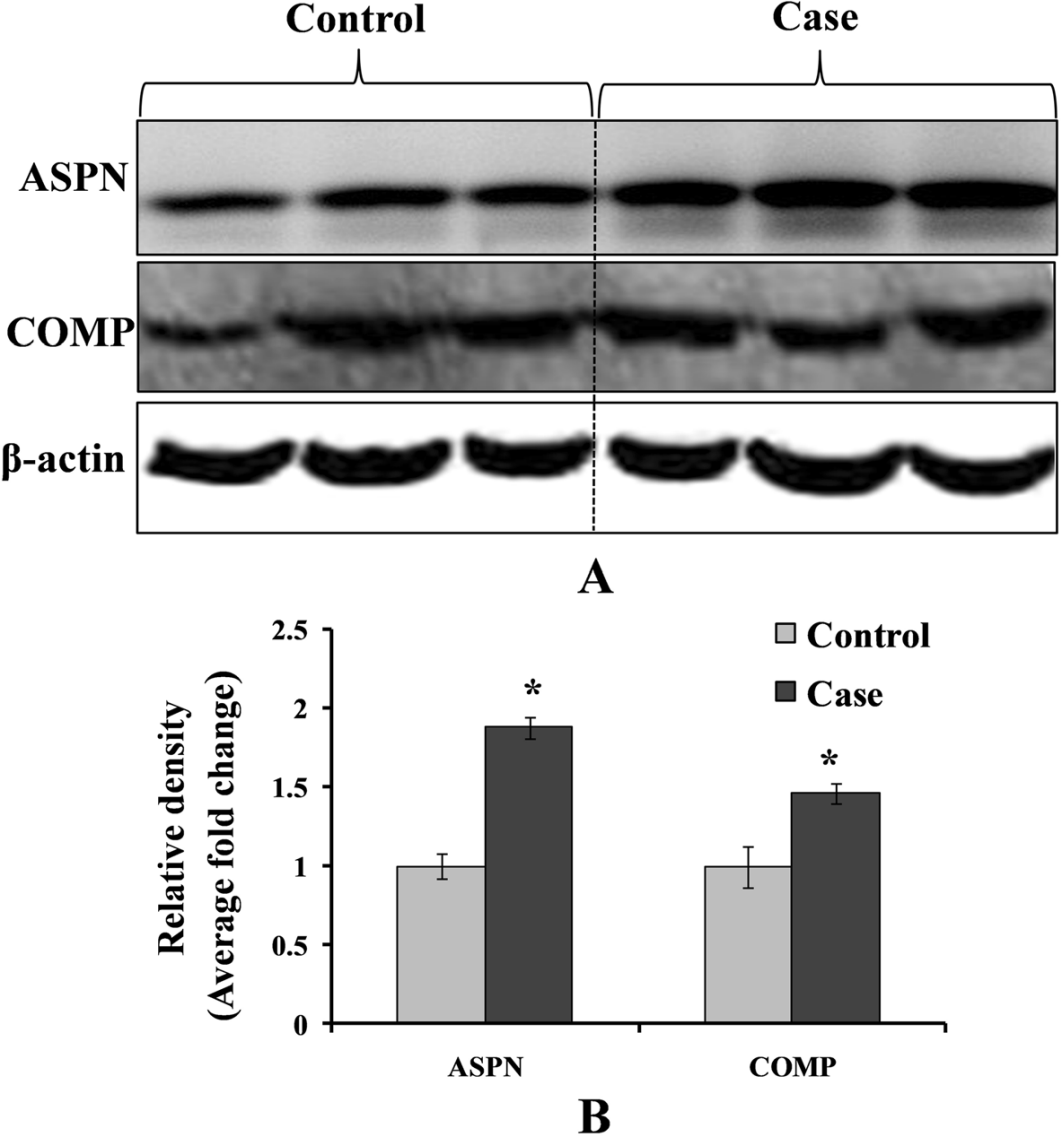
**a** Western blot analysis was performed to understand the protein level of ASPN and COMP in control and case study. **b** Bar diagram showing the relative protein density after normalization with β-actin. Relative protein density of ASPN and COMP was significantly increased in case study as compared to the control group. Representative blots showing three samples from each group (control and case). Values are expressed as mean ± SEM (*n* = 24). *p < 0.05 vs control

Immunoblotting studies in PBLs were done to determine the expression of these genes at the level of protein. Immunoblot analysis of freshly prepared lymphocyte, isolated from control, when incubated with polyclonal antibody raised against rabbit ASPN and COMP, demonstrated cross-reactivity with lymphocyte isolated from control. Densitometric analysis of the immunoblots revealed a significant increase in the immune reactivity of ASPN and COMP protein in controls (Fig. 2). Protein expression of ASPN was significantly increased (1.87-fold) in control subject after densitometry analysis with normalization of beta-actin. Similar to that observed in ASPN, immunoblot analysis revealed that expression of COMP (1.46-fold) protein was also found to be increased in lymphocyte. However, the magnitude of increase was relatively more in ASPN as compared to the COMP protein in blood lymphocytes.

## Discussion

Our data provides evidence that ASPN and COMP are polymorphic in the North Indian population. All of the selected SNPs, i.e., rs3739606 and rs331377, of ASPN gene were present in the North Indian population. The frequency of the variant allele (T) of rs 3739606 polymorphism of ASPN was found to be 26.9% in our study and is similar to the Caucasian and Oriental populations as reported in the literature [7]. Similarly, on gender-wise stratification, variant allele frequency was found to be similar in males and females in our population as reported in these two populations. The frequency of minor allele (G) of the polymorphism of ASPN (rs331377) was found to be 44.4% which is similar to that reported in the Caucasian population and relatively higher to the Oriental population [7].

One SNP as c279C/A was not found in the study population while this SNP was identified in the Japanese population [8]. This is in accordance with the literature, where it is reported that the frequency of variant allele A of the c279C/A, COMP polymorphism is very rare [8]. The frequency of variant allele of c279C/A polymorphism was found to be only 0.03% in the Japanese population [8, 23]. COMP (HPYCHIV) polymorphism was polymorphic in the Indian population. The frequency of T allele (3.7%) of HPYCH4IV in our population was similar to that reported in the Oriental and Caucasian populations. (http://www.ncbi.nlm.nih.gov/projects/SNP/snp_ref.cgi?rs=34467947).

Our case–control data have shown alterations in the distribution of variant alleles of ASPN in patients when compared to healthy controls. An increase in the frequency of variant T allele (rs 3739606) and G allele (rs 331377) was observed in knee OA patients compared to controls. As observed in Caucasians, increase in frequency of T allele of rs 3739606 was observed in our OA patients compared with controls. This association was not found to be significant on gender-wise stratification in our population as reported in Caucasians. In the cases with increased frequency of variant G allele (rs331377) of ASPN, no significant association to knee OA was observed [7]. This is similar to the previous case–control study in European subjects [24]. ASPN in knee OA susceptibility is very strong and is based solely on the functional properties of Asporin; Kizawa and Valdes tested ASPN for association with knee OA and observed a genetic association [11]. Further, in 2011, Shi indicated that ASPN is an important regulator in the development of knee OA [25]. However, the above study has not reported expression of either mRNA or the protein.

Little data is available on the functional activity of ASPN gene with knee OA, whereas there is no such study in the Indian population. A significant increase in mRNA and protein expression of ASPN in freshly prepared PBLs isolated from knee OA patients has further provided evidence that the expression of ASPN increases in late knee OA patients. Earlier, it was demonstrated by Kizawa and Valdes that Asporin is abundantly expressed in OA articular cartilage and that Asporin inhibits the expression of the genes encoding aggrecan and type II collagen, a major cartilage matrix gene through TGF-beta-mediated signaling [11]. In vitro binding assays showed a direct interaction between Asporin and TGF-beta [11]. Taken together, these findings provide another functional link between extracellular matrix proteins, TGF-beta activity, and chronic disease. A previous study in our laboratory has also reported reduced transcriptional activity of GDF-5 gene (member of TGF-beta family) in knee OA cases [17].

Our data have shown an increase in the frequency of T allele of HPYCH4IV polymorphism of COMP gene compared to controls; however, these data are not significantly associated. HPYCH4IV polymorphisms of COMP gene variant have already been reported in Caucasian family in PSACH and MED [26, 27]. Hereditary osteochondraldysplasias produce severe, early-onset OA and hence are models for common idiopathic OA [26]. Among them are pseudochondroplasia and multiple epiphyseal dysplasias, both of which are caused by mutations in the cartilage oligomeric matrix protein (COMP) gene [12]. Therefore, COMP may be a susceptible gene for knee OA. Mabuchi et al. screened all exons of the COMP gene with their flanking intron sequences and the promoter region for polymorphisms by direct sequencing [8]. Expression of COMP gene mRNA was upregulated in osteoarthritis cases. Increased level of COMP protein is also found in knee osteoarthritis cases in comparison with healthy control. COMP plays an important role in the structural integrity of cartilage via its interaction with other extracellular matrix proteins such as the collagens and fibronectin [14]. It can mediate the interaction of chondrocytes with the cartilage extracellular matrix through interaction with cell surface integrin receptors [14]. It is hypothesized that polymorphism may further increase the level of COMP, which may explain the elevated risk of OA in cases with variant genotype of COMP. Hence, it is reasonable to speculate that abnormal COMP, another major component of the cartilage matrix, could also cause knee OA. However, further studies are needed with a larger sample size to identify the possible role of this SNP in COMP in OA.

## Conclusion

Our study was focused on the functional role of genes in the pathogenesis of knee OA. RT-PCR and immunochemical studies demonstrated upregulation of ASPN and COMP in the PBLs isolated from knee OA patients, suggesting an involvement of these genes in cartilage disruption. Thus, this study demonstrates that polymorphism in ASPN and COMP genes is involved in the progression and susceptibility to the knee OA. Therefore, these genes could be used as a diagnostic marker for predicting knee osteoarthritis and identifying new therapeutic strategies for osteoarthritis.

## Data Availability

The datasets used and/or analyzed during the current study are available from the corresponding author on reasonable request.

## Abbreviations

ASPN: Asporin
COMP: Cartilage oligomeric matrix protein
KL grade: Kellgren–Lawrence Grading Scale
OA: Osteoarthritis
PBLs: Peripheral blood lymphocytes
VAS: Visual analog scale
WOMAC: The Western Ontario and McMaster Universities Osteoarthritis Index
